# Cyclosporine A for Recurrent Spontaneous Abortion: A Systematic Review and Meta-Analysis

**DOI:** 10.3390/biomedicines14091896

**Published:** 2026-08-25

**Authors:** Suning Huang, Yudi Song, Ang Cai, Yan Ning

**Affiliations:** Shenzhen Maternity and Child Healthcare Hospital, Women and Children’s Medical Center, Southern Medical University, Shenzhen 518000, China; carolsu2023@163.com (S.H.); songyudi8841@163.com (Y.S.); angela5639@163.com (A.C.)

**Keywords:** Cyclosporine A, recurrent spontaneous abortion, recurrent pregnancy loss, live birth, miscarriage, meta-analysis

## Abstract

**Objective**: To evaluate the efficacy and safety of cyclosporine A (CsA)-containing treatment for recurrent spontaneous abortion/recurrent pregnancy loss (RSA/RPL) through a systematic review and meta-analysis. **Methods**: PubMed, Embase, Cochrane Library, Web of Science, CNKI, Wanfang Database, and VIP were searched from inception to 31 December 2025. Randomized controlled trials and non-randomized controlled studies evaluating CsA-containing treatment in women with RSA/RPL were included. Risk ratios (RRs) with 95% confidence intervals (CIs) were pooled using a random-effects model. The main outcomes were live birth/successful delivery, pregnancy success/fetal protection success, miscarriage using the total enrolled population as the denominator, and maternal adverse events. The certainty of evidence was evaluated using the GRADE approach. **Results**: 13 studies involving 1181 participants were included. CsA-containing treatment was associated with a higher live birth/successful delivery rate than control treatment (RR = 1.32, 95% CI: 1.21–1.45). It was also associated with a higher pregnancy success/fetal protection success rate (RR = 1.34, 95% CI: 1.22–1.48) and a lower miscarriage rate (RR = 0.42, 95% CI: 0.33–0.54). No statistically significant difference in maternal adverse events was observed between the CsA-containing treatment and control groups (RR = 0.80, 95% CI: 0.43–1.49). The certainty of evidence was rated as low for efficacy outcomes and very low for maternal adverse events. **Conclusions**: CsA-containing treatment may be associated with improved pregnancy-related outcomes in women with RSA/RPL, including higher live birth/successful delivery and pregnancy success rates and a lower miscarriage rate. However, the certainty of evidence remains limited because of risk of bias, heterogeneous co-interventions, potential publication bias, and insufficient safety reporting. Further high-quality multicenter randomized controlled trials are needed.

## 1. Introduction

Recurrent spontaneous abortion (RSA) is a prevalent and distressing issue in reproductive medicine, significantly affecting the reproductive health and well-being of couples. Defined as the occurrence of two or more spontaneous abortions, RSA has an estimated incidence of 1–5% in the reproductive-age population [1,2,3]. The etiology of RSA is complex and multifactorial, involving genetic, anatomical, endocrine, immunological, and thrombophilic factors [4,5,6]. Despite extensive research, the cause remains unidentified in approximately 40–50% of cases, termed unexplained recurrent spontaneous abortion (URSA), which poses a great challenge to clinical treatment [7,8].

Immune-related factors play a crucial role in the pathogenesis of RSA. The maternal–fetal interface is a complex immunological microenvironment, and immune dysregulation can lead to rejection of the embryo, resulting in miscarriage [9]. Immunotherapy has emerged as a potential treatment option for RSA, aiming to modulate the immune system to support pregnancy. Among various immunotherapeutic agents, cyclosporine A (CsA) has drawn increasing attention in recent years. Structurally, CsA is a cyclic peptide with a lipophilic structure. In the context of URSA, CsA has been investigated for its potential role in improving pregnancy outcomes and modulating immune balance, including Th1/Th2 and Th17/Treg-related immune responses [10]. CsA has also been widely used in organ transplantation to prevent graft rejection and in the treatment of autoimmune diseases [10,11]. CsA, a potent immunosuppressive drug, has been widely used in organ transplantation to prevent graft rejection and in the treatment of autoimmune diseases [10,11]. Its mechanism of action mainly involves inhibiting the activation of T lymphocytes, thereby modulating the immune response [12]. In the context of RSA, CsA is hypothesized to regulate the immune environment at the maternal–fetal interface, promote the proliferation and invasion of trophoblast cells, and improve pregnancy outcomes [13,14].

Previous studies on the use of CsA in the treatment of RSA/RPL have reported potentially beneficial but not fully consistent findings. Some studies suggested that CsA-containing treatment may improve pregnancy-related outcomes in patients with RSA/RPL [15,16]. However, high-quality evidence from randomized controlled trials remains limited, and the use of CsA for RSA/RPL is not yet supported by international guidelines. These discrepancies may be attributed to differences in study design, patient populations, immune phenotypes, treatment regimens, background therapies, outcome definitions, and sample sizes.

Moreover, the safety of CsA during pregnancy remains an important concern. Although CsA has been used in pregnant women with organ transplants or autoimmune diseases with relatively acceptable outcomes in some cases [14], its potential maternal, fetal, and neonatal risks cannot be ignored [17]. Therefore, both efficacy and safety should be evaluated cautiously, particularly because adverse events, congenital anomalies, neonatal outcomes, and long-term offspring outcomes have not been consistently reported in existing RSA/RPL studies.

Although previous clinical studies and evidence syntheses have explored the potential role of CsA in RSA/RPL, uncertainty remains regarding the magnitude and certainty of its treatment effect, particularly for clinically important outcomes such as live birth or successful delivery. In addition, the interpretation of existing evidence is complicated by heterogeneous patient populations, variable CsA regimens, different background therapies, inconsistent outcome definitions, and limited safety reporting. Therefore, this systematic review and meta-analysis aimed to reassess the efficacy and safety of CsA-containing treatment in women with RSA/RPL, with particular emphasis on live birth/successful delivery, pregnancy success, miscarriage, maternal adverse events, sensitivity analyses, publication bias, and the certainty of evidence using the GRADE approach.

## 2. Materials and Methods

This systematic review and meta-analysis was conducted in accordance with the Preferred Reporting Items for Systematic Reviews and Meta-Analyses (PRISMA) statement (Table S1) [18]. This review was prospectively registered on PROSPERO, the registration number is CRD420261451811. As this study used data from previously published studies, ethical approval and patient informed consent were not required.

### 2.1. Literature Search

A comprehensive literature search was carried out across multiple databases, including PubMed, Embase, Cochrane Library, Web of Science, China National Knowledge Infrastructure (CNKI), Wanfang Database, and Chinese Scientific Journal Database (VIP). The search spanned from the inception of each database to 31 December 2025. The following core Boolean strategy was used and adapted for each database: (“Cyclosporine A” OR “cyclosporine” OR “ciclosporin” OR “CsA”) AND (“recurrent spontaneous abortion” OR “recurrent miscarriage” OR “habitual abortion” OR “recurrent pregnancy loss” OR “RSA” OR “RPL”). For Chinese databases, equivalent Chinese terms for cyclosporine A and recurrent spontaneous abortion/recurrent pregnancy loss were used. The search was limited to studies published in English or Chinese.

### 2.2. Inclusion and Exclusion Criteria

Studies were included if they met the following criteria: (1) the study design was a randomized controlled trial or a non-randomized controlled study; (2) the participants were women diagnosed with recurrent spontaneous abortion or recurrent pregnancy loss; (3) the intervention group received CsA-containing treatment, either alone or in combination with other therapies; (4) the control group received placebo, no treatment, or corresponding background treatment without CsA; and (5) the study reported at least one pregnancy-related or safety outcome, including live birth/successful delivery, pregnancy success/fetal protection success, miscarriage, maternal adverse events, or other relevant pregnancy outcomes.

Studies were excluded if they met any of the following criteria: (1) animal or in vitro studies only; (2) reviews, case reports, letters, editorials, conference abstracts, or duplicate publications; (3) studies not published in English or Chinese; (4) studies with insufficient data for extraction or quantitative analysis; (5) studies in which CsA was used in both the intervention and control groups; or (6) studies without relevant pregnancy or safety outcomes.

### 2.3. Literature Screening and Data Extraction

Two independent reviewers meticulously screened the retrieved studies based on the titles and abstracts. Studies that clearly did not meet the inclusion criteria were excluded at this stage. For the remaining potentially eligible studies, the full texts were retrieved and carefully evaluated. Any discrepancies between the reviewers were resolved through in-depth discussion or by consulting a third reviewer. Data extraction was performed using a pre-designed and standardized form, which included details such as the first author, publication year, country of origin, study design, sample size, patient characteristics, intervention details (type, dosage, duration, and administration route of Cyclosporine A), control group interventions, and outcome measures.

### 2.4. Assessment of Study Quality and Certainty of Evidence

The methodological quality of the included studies was rigorously assessed using established tools appropriate to each study design. For randomized controlled trials (RCTs), the Cochrane revised Risk of Bias tool (RoB 2) was employed, which assesses five domains: randomization process, deviations from intended interventions, missing outcome data, measurement of the outcome, and selection of the reported result. For non-randomized studies, the Newcastle–Ottawa Scale (NOS) [19] was used to assess quality across three domains: selection of participants, comparability of groups, and assessment of outcomes. Each item was rated accordingly, and an overall quality judgment was made. Studies with NOS scores ≥ 7 were considered high quality. All assessments were performed independently by two reviewers, with disagreements resolved by consensus or consultation with a third reviewer.

The certainty of evidence for the main outcomes was evaluated using the GRADE approach. The certainty of evidence was assessed across domains including risk of bias, inconsistency, indirectness, imprecision, and publication bias, and was rated as high, moderate, low, or very low.

### 2.5. Statistical Methods

Meta-analyses were performed using R software(Version 4.3.1) with the “meta” package. For dichotomous outcomes, risk ratios (RRs) with 95% confidence intervals (CIs) were calculated using the Mantel–Haenszel method under a random-effects model. Between-study variance was estimated using the DerSimonian–Laird method. A continuity correction of 0.5 was applied for studies with zero events in one treatment arm.

The main outcomes selected for quantitative synthesis were live birth/successful delivery, pregnancy success/fetal protection success, miscarriage using the total enrolled population as the denominator, and maternal adverse events. Heterogeneity was assessed using Cochran’s Q test and the I^2^ statistic. Given the limited number of studies available for each outcome and the low between-study heterogeneity observed in the primary analyses, formal subgroup meta-analyses were not performed because of the limited number of studies and heterogeneity of co-interventions. However, post hoc exploratory descriptive analyses were conducted according to background treatment and RSA/RPL subtype.

Publication bias was assessed using funnel plots, Egger’s regression test, and Begg’s rank correlation test when at least 10 studies were available for an outcome. Trim-and-fill analysis was further performed to explore the potential influence of small-study effects. A two-sided *p* value < 0.05 was considered statistically significant. In addition, post hoc exploratory descriptive analyses were performed according to whether CsA was combined with prednisone, whether CsA was combined with low molecular weight heparin, and whether the study population was classified as URSA or non-URSA. Because the number of studies in several subgroups was limited and co-interventions were heterogeneous, formal subgroup meta-analyses and tests for subgroup differences were not considered statistically reliable. Therefore, these findings were summarized descriptively.

## 3. Results

### 3.1. Study Selection and Characteristics

**Figure 1:**
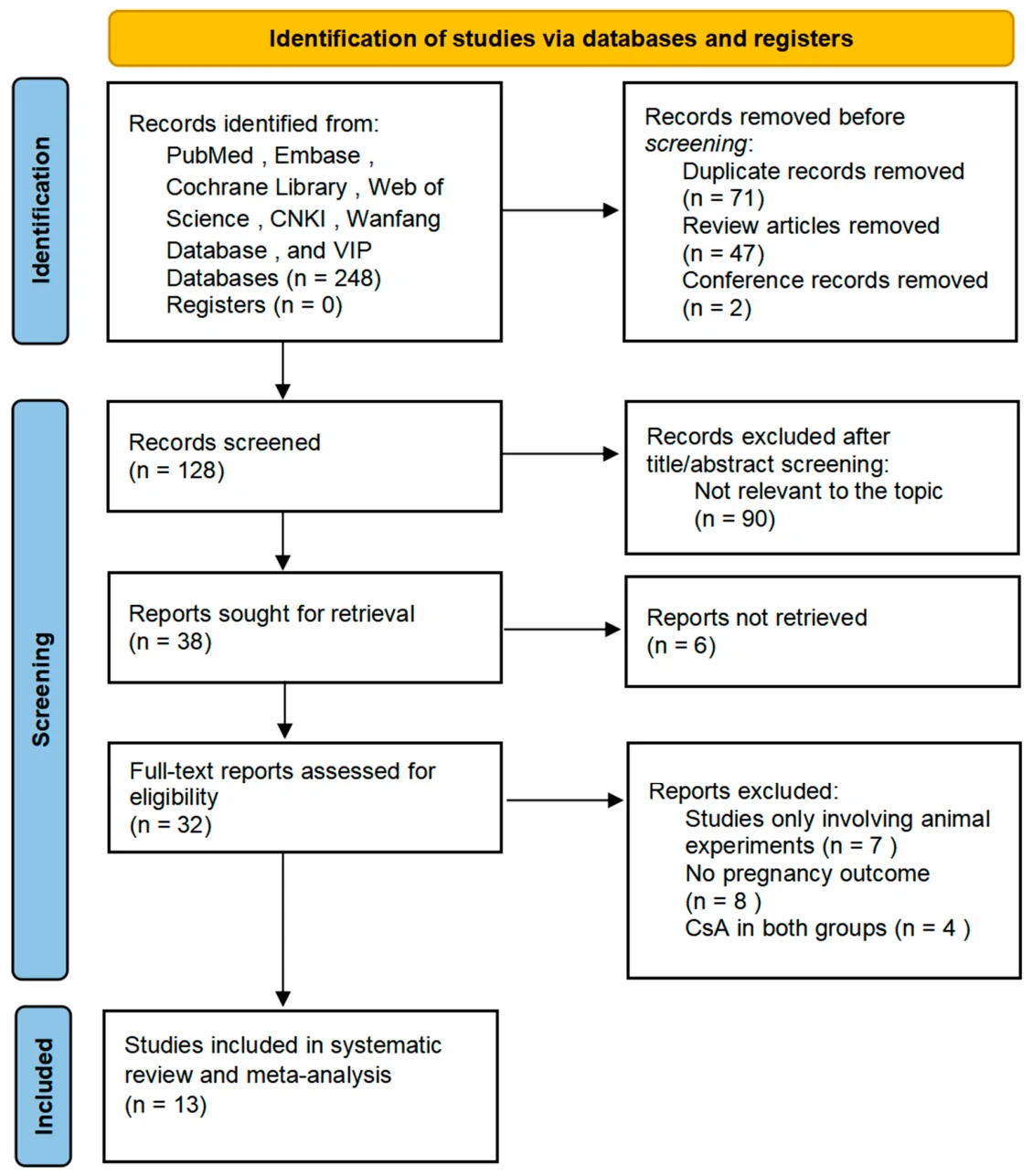
PRISMA 2020 flow diagram for the identification of studies included in the systematic review and meta-analysis.

The study selection process is shown in Figure 1. A total of 248 records were initially identified from the databases. Before screening, 71 duplicate records, 47 review articles, and 2 conference records were removed. After title and abstract screening, 90 records were excluded because they were not relevant to the topic. Thirty-eight full-text reports were sought for retrieval, of which 6 could not be retrieved. Finally, 32 full-text reports were assessed for eligibility. Among these, 7 animal studies, 8 studies without pregnancy outcomes, and 4 studies in which CsA was used in both groups were excluded. Ultimately, 13 studies were included in this systematic review and meta-analysis.

The characteristics of the included studies are summarized in Table 1. The 13 included studies involved 1181 participants. Ten studies were randomized controlled trials, and three were non-randomized controlled studies. Most studies were conducted in China, and one study was conducted in Iran. The included populations varied across studies, including unexplained RSA, immune-related RSA, RSA with increased natural killer cells, RPL with elevated Th1/Th2 ratio, and RSA with negative blocking antibody. CsA was used either alone or in combination with background treatments, including dydrogesterone, progesterone, prednisone, low molecular weight heparin, traditional Chinese medicine, or lymphocyte immunotherapy. The CsA dose, route, treatment duration, and control interventions varied across studies.

### 3.2. Efficacy and Safety of CsA-Containing Treatment in RSA/RPL

#### 3.2.1. Live Birth/Successful Delivery

**Figure 2:**
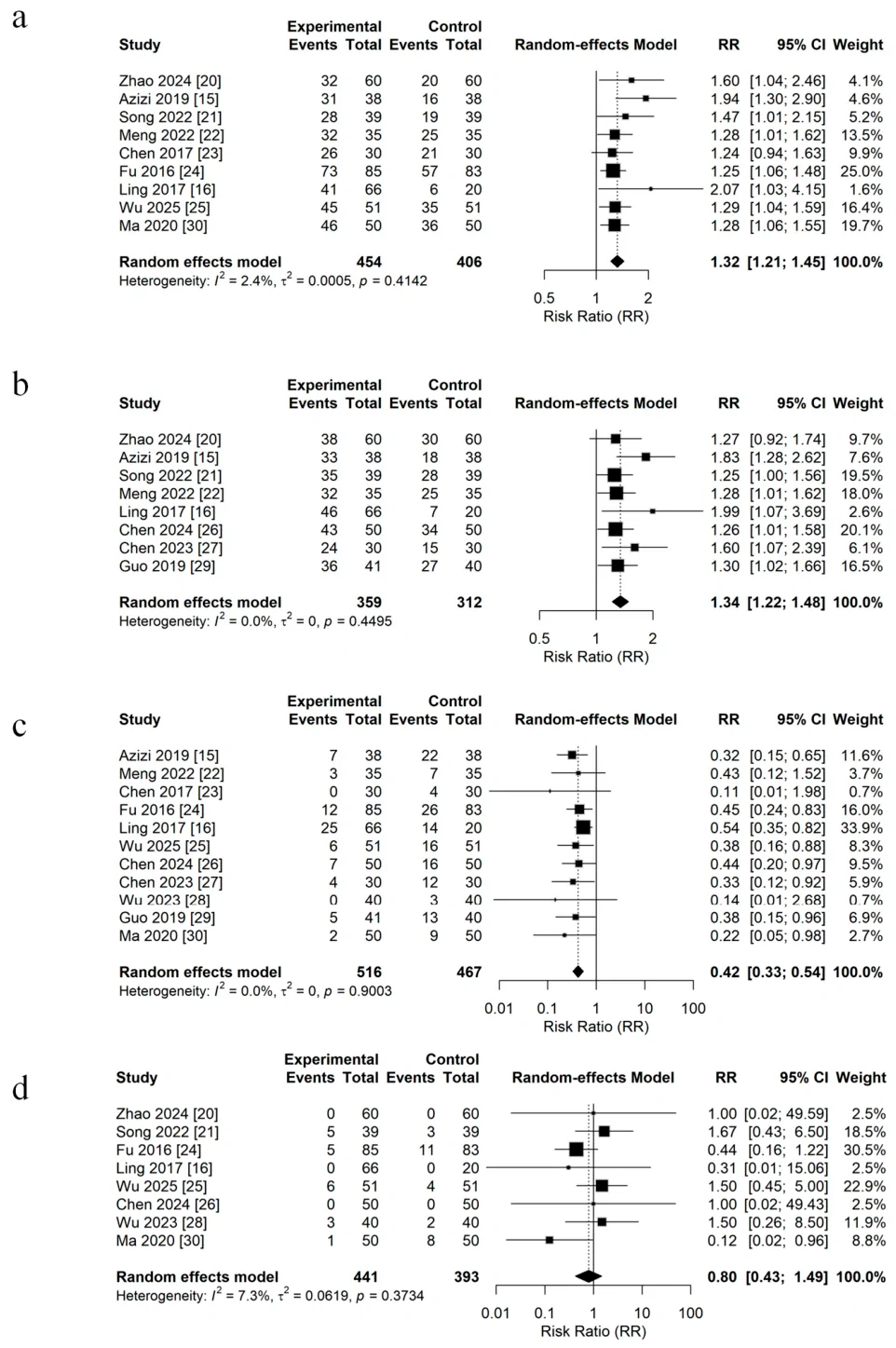
Forest plots of the effects of CsA-containing treatment on pregnancy-related outcomes. (**a**) Live birth/successful delivery; (**b**) pregnancy success/fetal protection success; (**c**) miscarriage using the total enrolled population as the denominator; (**d**) maternal adverse events.

A total of 9 studies reported live birth or successful delivery outcomes, involving 860 participants, including 454 in the CsA-containing treatment group and 406 in the control group. The pooled analysis showed that CsA-containing treatment was associated with a higher live birth/successful delivery rate compared with control treatment (RR = 1.32, 95% CI: 1.21–1.45; Figure 2a). Low heterogeneity was observed across studies (I^2^ = 2.4%, *p* = 0.4142).

#### 3.2.2. Pregnancy Success/Fetal Protection Success

8 studies reported pregnancy success or fetal protection success, involving 671 participants, including 359 in the CsA-containing treatment group and 312 in the control group. The pooled result indicated that CsA-containing treatment was associated with a higher pregnancy success rate than control treatment (RR = 1.34, 95% CI: 1.22–1.48; Figure 2b). No heterogeneity was observed among the included studies (I^2^ = 0.0%, *p* = 0.4495).

#### 3.2.3. Miscarriage

In total, 11 studies reported miscarriage outcomes using the total enrolled population as the denominator, involving 983 participants, including 516 in the CsA-containing treatment group and 467 in the control group. The pooled analysis showed that CsA-containing treatment was associated with a lower miscarriage rate compared with control treatment (RR = 0.42, 95% CI: 0.33–0.54; Figure 2c). No heterogeneity was observed across studies (I^2^ = 0.0%, *p* = 0.9003).

#### 3.2.4. Maternal Adverse Events

A total of 8 studies reported maternal adverse events, involving 834 participants, including 441 in the CsA-containing treatment group and 393 in the control group. The pooled analysis showed no statistically significant difference in maternal adverse events between the CsA-containing treatment group and the control group (RR = 0.80, 95% CI: 0.43–1.49; Figure 2d). Low heterogeneity was observed (I^2^ = 7.3%, *p* = 0.3734). However, this result should be interpreted cautiously because adverse events were inconsistently reported, and several studies had zero events in both arms.

### 3.3. Sensitivity Analyses

**Figure 3:**
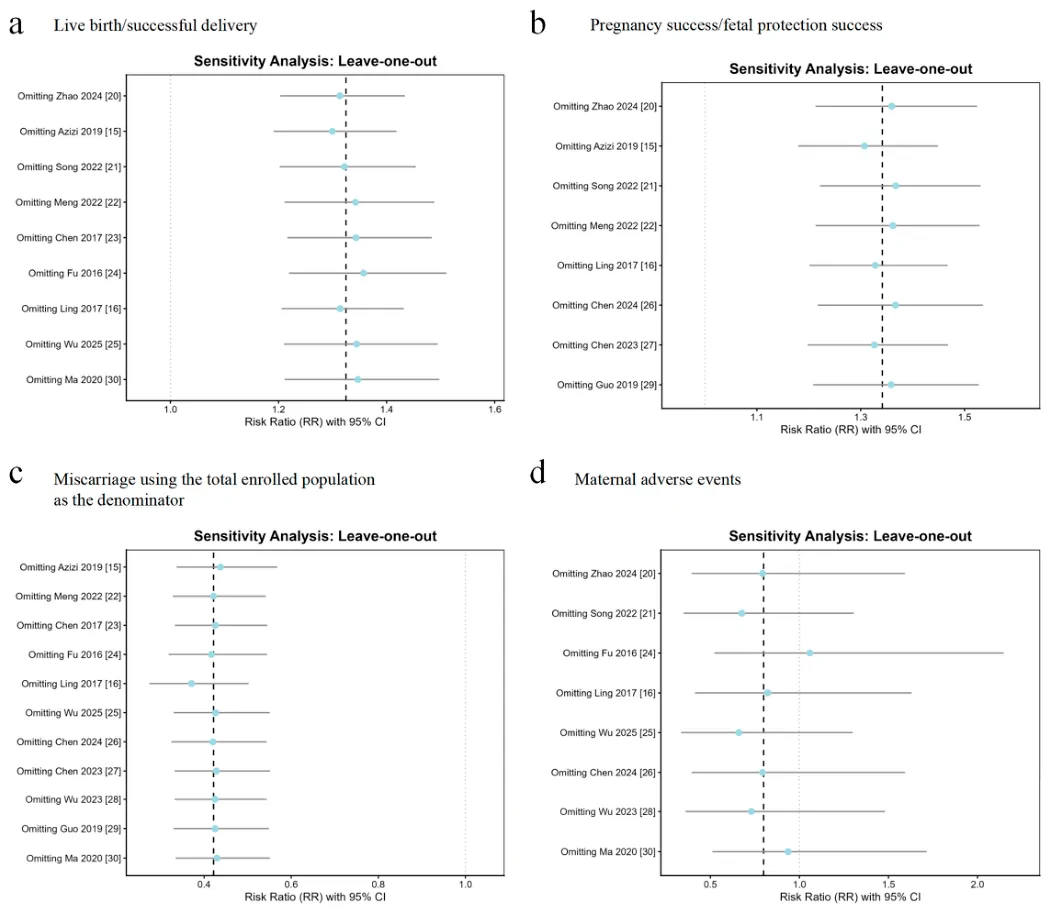
Leave-one-out sensitivity analyses of the effects of CsA-containing treatment. (**a**) Live birth/successful delivery; (**b**) pregnancy success/fetal protection success; (**c**) miscarriage using the total enrolled population as the denominator; (**d**) maternal adverse events.

Leave-one-out sensitivity analyses showed that the pooled estimates for live birth/successful delivery, pregnancy success/fetal protection success, miscarriage, and maternal adverse events remained directionally consistent after omitting each study in turn, indicating that no single study had a substantial influence on the overall findings (Figure 3).

**Figure 4:**
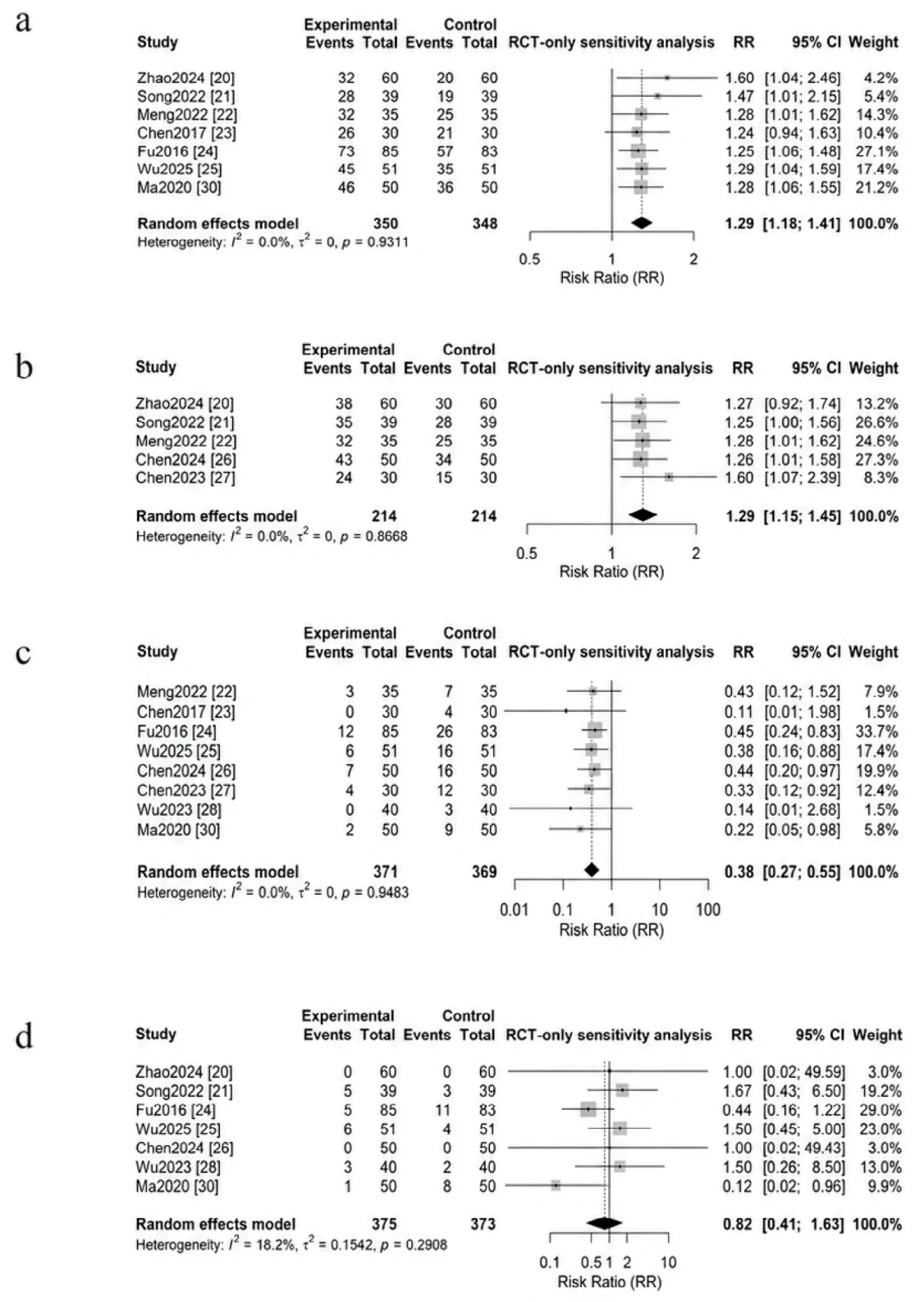
RCT-only sensitivity analyses of the effects of CsA-containing treatment. (**a**) Live birth/successful delivery; (**b**) pregnancy success/fetal protection success; (**c**) miscarriage using the total enrolled population as the denominator; (**d**) maternal adverse events.

Sensitivity analyses restricted to randomized controlled trials yielded results consistent with the primary analyses (Figure 4). In the RCT-only analysis, CsA-containing treatment remained associated with higher live birth/successful delivery (RR = 1.29, 95% CI: 1.18–1.41; I^2^ = 0.0%) and pregnancy success/fetal protection success (RR = 1.29, 95% CI: 1.15–1.45; I^2^ = 0.0%). The reduction in miscarriage also remained significant (RR = 0.38, 95% CI: 0.27–0.55; I^2^ = 0.0%). For maternal adverse events, the RCT-only analysis showed no statistically significant difference between groups (RR = 0.82, 95% CI: 0.41–1.63; I^2^ = 18.2%).

### 3.4. Risk of Bias Assessment

The Newcastle–Ottawa Scale was used to assess the quality of non-randomized studies. The total scores ranged from 7 to 8, indicating moderate to high methodological quality (Table 2). For randomized controlled trials, risk of bias was assessed using RoB 2.0. Two studies were judged as having a low overall risk of bias, whereas the remaining randomized trials were judged as having some concerns. These concerns were mainly related to insufficient information regarding the randomization process, allocation concealment, or deviations from intended interventions. To ensure transparency, the detailed domain-level RoB 2.0 judgments for each randomized controlled trial are retained in Table 3.

### 3.5. Publication Bias Assessment

**Figure 5:**
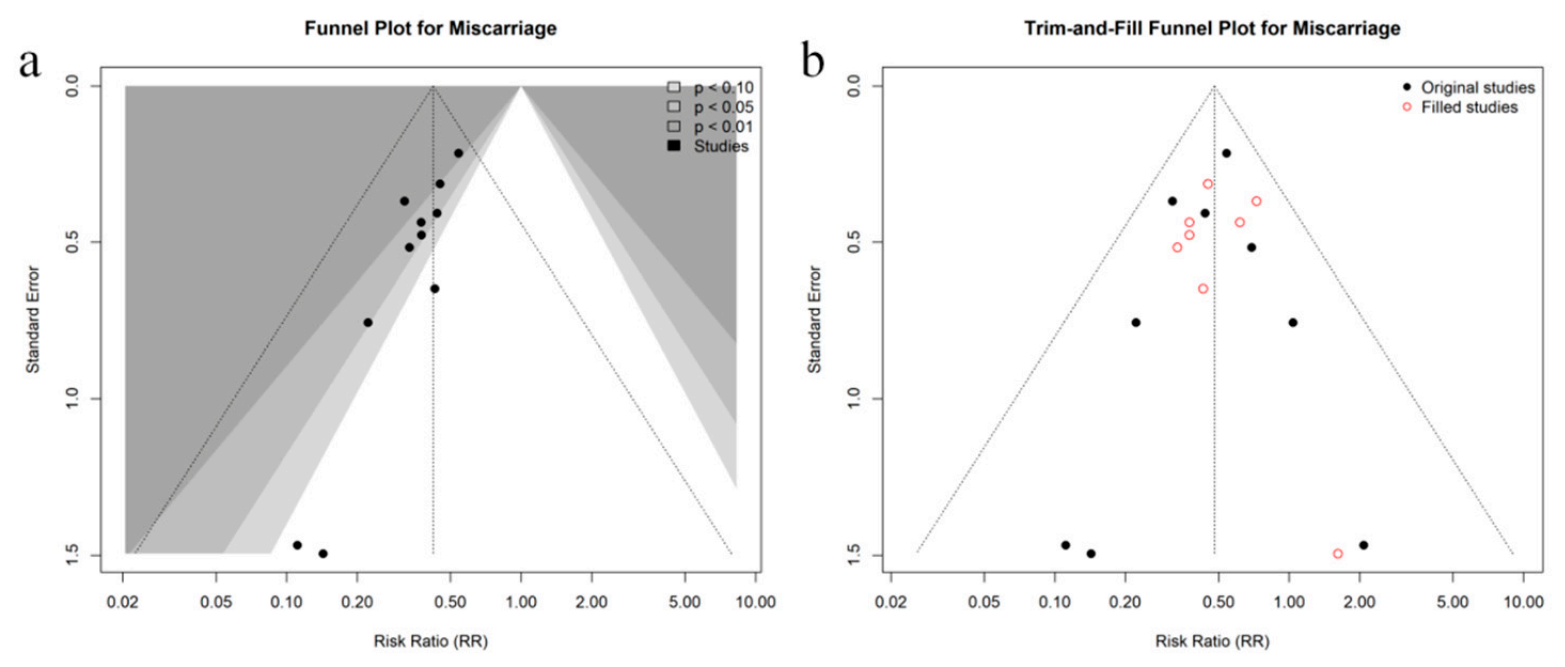
Publication bias assessment for miscarriage. (**a**) Funnel plot; (**b**) trim-and-fill funnel plot.

Because miscarriage was the only outcome reported by at least 10 studies, formal publication bias assessment was performed for this outcome. The funnel plot appeared asymmetric (Figure 5a), and both Egger’s regression test (*p* = 0.0006) and Begg’s rank correlation test (*p* = 0.0158) suggested potential publication bias. Trim-and-fill analysis imputed six potentially missing studies (Figure 5b), yielding an adjusted pooled RR of 0.48 (95% CI: 0.39–0.60), compared with the original pooled RR of 0.42 (95% CI: 0.33–0.54). Although the adjusted estimate remained statistically significant, the attenuation of the effect size indicates that the miscarriage outcome should be interpreted cautiously.

### 3.6. Certainty of Evidence

The certainty of evidence was evaluated using the GRADE approach. The certainty of evidence was rated as low for live birth/successful delivery, pregnancy success/fetal protection success, and miscarriage, mainly because of risk of bias, indirectness, or publication bias. The certainty of evidence for maternal adverse events was rated as very low because of risk of bias, serious imprecision, and incomplete safety reporting. The GRADE summary of findings is presented in Table 4.

### 3.7. Exploratory Descriptive Analyses According to Background Treatment and RSA/RPL Subtype

Post hoc exploratory descriptive analyses were conducted according to background treatment and RSA/RPL subtype. Two studies used CsA in combination with prednisone, involving 222 participants, whereas 11 studies did not include prednisone in the CsA-containing regimen, involving 959 participants. Two studies used CsA in combination with low molecular weight heparin, involving 180 participants, whereas 11 studies did not include low molecular weight heparin, involving 1001 participants. Regarding RSA/RPL subtype, eight studies included URSA populations, involving 635 participants, whereas five studies included non-URSA populations, involving 546 participants.

Only a small number of studies evaluated CsA-containing regimens combined with prednisone or low molecular weight heparin. In addition, background treatments varied substantially across studies, including dydrogesterone, progesterone, prednisone, low molecular weight heparin, traditional Chinese medicine, and lymphocyte immunotherapy. Therefore, formal subgroup meta-analyses and tests for subgroup differences were not considered statistically reliable. These findings were summarized descriptively in Table 5 and should be interpreted as exploratory evidence regarding CsA-containing regimens rather than the independent effect of CsA alone.

## 4. Discussion

This systematic review and meta-analysis evaluated the efficacy and safety of CsA-containing treatment in women with RSA/RPL. Compared with control treatment, CsA-containing regimens were associated with higher live birth/successful delivery and pregnancy success/fetal protection success rates, as well as a lower miscarriage rate. These findings remained broadly consistent in leave-one-out sensitivity analyses and in analyses restricted to randomized controlled trials, suggesting that the main efficacy findings were not driven by a single study or by the inclusion of non-randomized studies. However, the certainty of evidence was rated as low for the efficacy outcomes, and these results should therefore be interpreted cautiously.

CsA is a potent immunosuppressive agent that has been widely used to prevent graft rejection and graft-versus-host disease in organ and tissue transplantation [31]. Its mechanism involves binding to high-affinity protein receptors on T lymphocytes, thereby inhibiting mRNA transcription and suppressing interleukin-2 production and receptor expression [32]. Given the substantial role of immune dysregulation in RSA/RPL [33], CsA has been explored as a potential therapy for immune-mediated pregnancy loss. However, current clinical guidelines do not recommend the routine use of CsA for RSA/RPL management because of insufficient evidence regarding its efficacy and safety [34]. Therefore, the findings of this meta-analysis should be considered hypothesis-supporting rather than definitive evidence for routine clinical use.

The potential benefit of CsA in RSA/RPL may be biologically plausible. The potential benefit of CsA in RSA/RPL may be biologically plausible. Mechanistically, CsA exerts its immunosuppressive effects mainly through the cyclophilin–calcineurin–nuclear factor of activated T cells (NFAT) pathway. After entering cells, CsA binds to cyclophilin to form a CsA–cyclophilin complex, which inhibits calcineurin activity. This prevents NFAT dephosphorylation and nuclear translocation, thereby reducing interleukin-2 transcription and suppressing T-cell activation and proliferation [31,32]. In the context of RSA/RPL, immune dysregulation at the maternal–fetal interface, including excessive Th1-type immune activation, abnormal Th1/Th2 balance, increased natural killer cell activity, and impaired maternal–fetal immune tolerance, has been implicated in pregnancy loss. By suppressing aberrant T-cell activation and inflammatory cytokine production, CsA may help restore a more tolerogenic immune microenvironment at the maternal–fetal interface. In addition, experimental studies suggest that CsA may protect trophoblasts from oxidative injury and apoptosis [35], and animal studies have shown that CsA may improve pregnancy outcomes by promoting trophoblast function and inducing maternal tolerance to the allogeneic fetus [36]. These mechanisms may partly explain the observed improvement in pregnancy-related outcomes, particularly among patients with immune-related RSA/RPL. However, whether these mechanisms fully explain the clinical benefits observed in RSA/RPL remains uncertain and requires further mechanistic and clinical investigation. Immune dysregulation, including excessive Th1-type immune activation, abnormal Th1/Th2 balance, increased natural killer cell activity, and impaired maternal–fetal immune tolerance, has been implicated in a subset of patients with RSA/RPL. CsA may help modulate the immune microenvironment at the maternal–fetal interface by suppressing aberrant T-cell activation and inflammatory responses. Mechanistic studies suggest that CsA may protect trophoblasts from oxidative injury [35]. In addition, animal studies have shown that CsA may improve pregnancy outcomes by promoting trophoblast function and inducing maternal tolerance to the allogeneic fetus [36]. These mechanisms may partly explain the observed improvement in pregnancy-related outcomes, particularly among patients with immune-related RSA/RPL.

Nevertheless, several factors limit the interpretation of the pooled efficacy estimates. First, the included studies varied in patient selection, RSA/RPL definitions, immune phenotypes, CsA dosage, treatment duration, and background therapies. In many studies, CsA was used in combination with other treatments, such as dydrogesterone, prednisone, low molecular weight heparin, traditional Chinese medicine, or lymphocyte immunotherapy. Therefore, the pooled effects should be interpreted as the effects of CsA-containing regimens rather than the independent effect of CsA alone. The post hoc exploratory descriptive analyses further showed that only a small number of studies evaluated CsA-containing regimens combined with prednisone or low molecular weight heparin, whereas most studies used heterogeneous background treatments. Therefore, the contribution of background therapies could not be reliably quantified using the available aggregate study-level data. Although the observed effects may partly reflect the immunomodulatory role of CsA, they may also be influenced by concomitant treatments such as progesterone, dydrogesterone, prednisone, low molecular weight heparin, traditional Chinese medicine, or lymphocyte immunotherapy. Future randomized controlled trials should use standardized background therapy or factorial designs to distinguish the independent and additive effects of CsA from those of concomitant treatments. For example, factorial-design trials comparing CsA versus no CsA and prednisone or low molecular weight heparin versus no such treatment would help clarify the specific contribution of CsA-containing regimens. Second, recurrent miscarriage is associated with subsequent adverse maternal and pregnancy outcomes, and differences in baseline risk profiles may influence outcome interpretation [37]. Third, some outcomes were not uniformly defined across studies. For example, live birth, successful delivery, delivery of a healthy infant, ongoing pregnancy, and fetal protection success were reported using different terms and definitions. To reduce misclassification, we separated live birth/successful delivery from pregnancy success/fetal protection success and analyzed miscarriage only when the total enrolled population could be used as the denominator. The absence of statistical heterogeneity in the miscarriage analysis may be partly related to the use of a consistent denominator and the similar direction of effect across studies; however, this should not be interpreted as evidence of no clinical heterogeneity, given differences in patient populations, CsA regimens, background therapies, and comparator treatments.

Safety evidence remains limited. The pooled analysis did not show a statistically significant increase in maternal adverse events in the CsA-containing treatment group. However, this finding should not be interpreted as confirming the safety of CsA during pregnancy. In one included cohort study, Ling et al. reported that low-dose CsA treatment was not associated with obvious maternal adverse reactions, intrauterine growth restriction, or congenital anomalies [16]. In addition, previous evidence from pregnant patients with systemic autoimmune diseases has suggested that CsA may be used in selected clinical contexts [38]. Nevertheless, adverse events were inconsistently reported across the included studies, several studies had zero events in both arms, and fetal, neonatal, congenital anomaly, and long-term offspring outcomes were rarely or incompletely assessed. Several safety outcomes were planned for extraction but could not be quantitatively synthesized because of insufficient or inconsistent reporting. These included maternal renal dysfunction, hepatic dysfunction, hypertension, infection, gastrointestinal symptoms, treatment discontinuation, fetal growth restriction, congenital anomalies, preterm birth, birth weight, Apgar score, neonatal complications, neonatal intensive care unit admission, and long-term offspring outcomes. Moreover, experimental studies have raised concerns regarding potential developmental toxicity at specific concentrations [39]. Therefore, future studies should include standardized maternal, fetal, neonatal, and long-term safety outcomes with adequate follow-up.

This study has several strengths, including the prioritization of live birth/successful delivery as a clinically important outcome, separation of pregnancy success-related outcomes, distinction of miscarriage denominators, assessment of maternal adverse events, sensitivity analyses, publication bias assessment, and GRADE evaluation. However, several limitations should be acknowledged. Most included studies were conducted in China, with only one study from Iran, which may limit generalizability. Some studies had methodological limitations, including unclear randomization procedures, limited allocation concealment, and insufficient blinding. In addition, sample sizes were generally small, co-interventions were frequently used, and safety outcomes were inconsistently reported, making it difficult to isolate the independent effect of CsA and to draw firm conclusions regarding maternal, fetal, and neonatal safety.

## 5. Conclusions

This systematic review and meta-analysis suggests that CsA-containing treatment may be associated with improved pregnancy-related outcomes in women with RSA/RPL, including higher live birth/successful delivery and pregnancy success/fetal protection success rates, as well as a lower miscarriage rate. No statistically significant increase in maternal adverse events was observed; however, the available safety evidence remains insufficient because adverse events and fetal, neonatal, and long-term offspring outcomes were inconsistently reported. Given the low certainty of evidence for efficacy outcomes, very low certainty of evidence for maternal adverse events, and the predominance of studies conducted in China, these findings should be interpreted cautiously and may have limited global generalizability. Therefore, current evidence does not support the routine or indiscriminate use of CsA in all patients with RSA/RPL. Further well-designed, adequately powered, multicenter randomized controlled trials with standardized diagnostic criteria, immune phenotyping, uniform CsA regimens, appropriate control groups, and comprehensive maternal–fetal safety assessments are needed to confirm the efficacy and clarify the safety profile of CsA-containing treatment during pregnancy.

## Figures and Tables

**Table 1:** Study characteristics.

Author (Year)	Country	Study Design	RSA Type	Sample Size, CsA/Control	Previous Miscarriage/ Pregnancy History *		Age		Intervention	Control	CsA Dose
					Intervention	Control	Intervention	Control			
Zhao et al. (2024) [20]	China	RCT	Immune-related RSA	60/60	2.800 ± 0.971	3.100 ± 1.131	33.368 ± 3.795	32.083 ± 4.291	CsA + aspirin + prednisone + low molecular weight heparin	Aspirin + prednisone + LMWH	100–150 mg/day, oral; duration as reported in original protocol
Azizi et al. (2019) [15]	Iran	Controlled clinical study	RPL with elevated Th1/Th2 ratio	38/38	4.1 ± 1.2	4.4 ± 1.2	31.8 ± 3.2	32.1 ± 3.3	CsA	No treatment	50 mg three times daily, oral; for 6 months after positive β-HCG
Song et al. (2022) [21]	China	RCT	URSA	39/39	4.55 ± 0.37	4.52 ± 0.33	29.92 ± 2.48	29.86 ± 2.45	CsA + dydrogesterone	Dydrogesterone	50 mg three times daily, oral; for 4 weeks
Meng (2022) [22]	China	RCT	URSA	35/35	4.34 ± 1.09	4.2 ± 1.15	32.88 ± 4.23	33.29 ± 4.40	CsA + dydrogesterone + Shoutai Pill	Dydrogesterone + Shoutai Pill	50 mg twice daily, oral; until 12 weeks of gestation
Chen et al. (2017) [23]	China	RCT	Refractory URSA	30/30	4.65 ± 1.34	4.12 ± 1.21	31.01 ± 4. 42	30.12 ± 4.36	CsA + progesterone + HCG	Progesterone + HCG	30 mg/day, oral; started before pregnancy and adjusted according to antibody status
Fu et al. (2016) [24]	China	Randomized, single-blind, placebo-controlled trial	Refractory immune-related RSA	85/83	5.5 ± 0.3	5.4 ± 0.4	34.8 ± 2.7	33.9 ± 2.8	CsA + folic acid + progesterone + IVIG + paternal lymphocyte immunotherapy	Placebo + folic acid + progesterone + IVIG + LIT	2–4 mg/kg twice daily, oral; total 4–8 mg/kg/day; trough concentration maintained at 80–150 mg/L
Ling et al. (2017) [16]	China	Prospective non-RCT	URSA	66/20	3.43 ± 1.37	2.63 ± 0.68	32.90 ± 5.01	30.45 ± 4.51	CsA	Progesterone	100 mg/day, oral; for 30 days after positive pregnancy test
Wu et al. (2025) [25]	China	RCT	RSA with negative blocking antibody	51/51	3.12 ± 0.41	3.04 ± 0.38	29.67 ± 4.14	30.11 ± 4.05	CsA + prednisone + progesterone + HCG + Elevit	Progesterone + HCG + Elevit	50 mg two to three times daily, oral; blood concentration maintained at 80–150 ng/mL; until 12 weeks of gestation
Chen et al. (2024) [26]	China	Prospective RCT	URSA	50/50	2.38 ± 0.53	2.26 ± 0.60	30.70 ± 4.14	29.86 ± 4.05	CsA + dydrogesterone	Dydrogesterone	150 mg/day, oral; discontinued after ultrasound detection of fetal heartbeat
Chen et al. (2023) [27]	China	RCT	URSA	30/30	3.17 ± 0.91	3.13 ± 0.82	28.7 ± 4.18	29.23 ± 4.47	CsA + low molecular weight heparin calcium	LMWH calcium	50 mg every 8 h, oral; blood concentration monitored and adjusted
Wu et al. (2023) [28]	China	RCT	Early RSA	40/40	3.91 ± 0.51	4.02 ± 0.48	28.65 ± 2.10	29.31 ± 2.15	CsA soft capsules + Zishen Yutai Pill	Zishen Yutai Pill	50 mg every 8 h, oral; for 16 days
Guo et al. (2019) [29]	China	Non-randomized controlled study	URSA	41/40	3.51 ± 1.08	3.12 ± 1.01	31.25 ± 4.05	30.31 ± 3.89	CsA + dydrogesterone	Dydrogesterone	50 mg three times daily, oral; for 4 weeks; blood concentration monitored
Ma et al. (2020) [30]	China	RCT	URSA	50/50	4.2 ± 1.1	4.3 ± 1.3	28.1 ± 5.6	28.0 ± 6.9	CsA + lymphocyte immunotherapy	LIT	2-4 mg/kg/day, oral; duration as reported in original study

Abbreviations: CsA, cyclosporine A; HCG, human chorionic gonadotropin; IVIG, intravenous immunoglobulin; LIT, lymphocyte immunotherapy; LMWH, low molecular weight heparin; RCT, randomized controlled trial; RPL, recurrent pregnancy loss; RSA, recurrent spontaneous abortion; URSA, unexplained recurrent spontaneous abortion. * Values were extracted as reported in the original studies; some studies reported a previous miscarriage number, whereas others reported pregnancy frequency or abortion frequency.

**Table 2:** Newcastle–Ottawa Scale assessment of non-randomized studies.

Study	Selection	Comparability	Outcome	Total Score
Azizi 2019 [15]	4	1	3	8
Ling 2017 [16]	4	1	3	8
Guo 2019 [29]	3	1	3	7

**Table 3:** Risk of bias assessment of included randomized controlled trials (RoB 2.0).

Study	D1: Randomization Process	D2: Deviations from Intended Interventions	D3: Missing Outcome Data	D4: Measurement of the Outcome	D5: Selection of the Reported Result	Overall Risk of Bias
Zhao 2024 [20]	Some concerns	Some concerns	Low	Low	Low	Some concerns
Song 2022 [21]	Some concerns	Some concerns	Low	Low	Low	Some concerns
Meng 2022 [22]	Some concerns	Some concerns	Low	Low	Low	Some concerns
Chen 2017 [23]	Some concerns	Some concerns	Low	Low	Low	Some concerns
Wu 2025 [25]	Some concerns	Some concerns	Low	Low	Low	Some concerns
Chen 2024 [26]	Some concerns	Some concerns	Low	Low	Low	Some concerns
Chen 2023 [27]	Some concerns	Some concerns	Low	Low	Low	Some concerns
Wu 2023 [28]	Some concerns	Some concerns	Low	Low	Low	Some concerns
Ma 2020 [30]	Low	Low	Low	Low	Low	Low
Fu 2016 [24]	Low	Low	Low	Low	Low	Low

**Table 4:** Summary of findings and certainty of evidence using GRADE.

Outcome	No. of Studies	Participants	Effect Estimate	Certainty of Evidence	Reasons for Downgrading
Live birth/successful delivery	9	860	RR = 1.32, 95% CI: 1.21–1.45	Low	Downgraded for risk of bias and indirectness. Several studies had some concerns regarding randomization, allocation concealment, or blinding, and the outcome definitions included live birth, successful delivery, or delivery of a healthy infant.
Pregnancy success/fetal protection success	8	671	RR = 1.34, 95% CI: 1.22–1.48	Low	Downgraded for risk of bias and indirectness. The definition of pregnancy success varied across studies, including ongoing pregnancy, fetal protection success, or successful pregnancy maintenance.
Miscarriage	11	983	RR = 0.42, 95% CI: 0.33–0.54	Low	Downgraded for risk of bias and publication bias. Funnel plot asymmetry and Egger’s and Begg’s tests suggested potential publication bias, although the trim-and-fill analysis did not change the direction of the effect.
Maternal adverse events	8	834	RR = 0.80, 95% CI: 0.43–1.49	Very low	Downgraded for risk of bias, serious imprecision, and incomplete safety reporting. Several studies reported zero events in both arms, and fetal, neonatal, and long-term offspring safety outcomes were rarely reported.

**Table 5:** Summary of post hoc exploratory descriptive analyses.

Subgroup Factor	Category	Included Studies	No. of Studies	Participants, CsA/Control	Total Participants
Prednisone combination	CsA combined with prednisone	Zhao 2024 [20]; Wu 2025 [25]	2	111/111	222
Prednisone combination	CsA not combined with prednisone	Azizi 2019 [15]; Song 2022 [21]; Meng 2022 [22]; Chen 2017 [23]; Fu 2016 [24]; Ling 2017 [16]; Chen 2024 [26]; Chen 2023 [27]; Wu 2023 [28]; Guo 2019 [29]; Ma 2020 [30]	11	504/455	959
LMWH combination	CsA combined with LMWH	Zhao 2024 [20]; Chen 2023 [27]	2	90/90	180
LMWH combination	CsA not combined with LMWH	Azizi 2019 [15]; Song 2022 [21]; Meng 2022 [22]; Chen 2017 [23]; Fu 2016 [24]; Ling 2017 [16]; Wu 2025 [25]; Chen 2024 [26]; Wu 2023 [28]; Guo 2019 [29]; Ma 2020 [30]	11	525/476	1001
RSA/RPL subtype	URSA	Song 2022 [21]; Meng 2022 [22]; Chen 2017 [23]; Ling 2017 [16]; Chen 2024 [26]; Chen 2023 [27]; Guo 2019 [29]; Ma 2020 [30]	8	341/294	635
RSA/RPL subtype	Non-URSA	Zhao 2024 [20]; Azizi 2019 [15]; Fu 2016 [24]; Wu 2025 [25]; Wu 2023 [28]	5	274/272	546

## Data Availability

The datasets used and/or analyzed during the current study are available from the corresponding author upon request.

## Supplementary Materials

The following supporting information can be downloaded at: https://www.mdpi.com/article/10.3390/biomedicines14091896/s1, Table S1: PRISMA 2020 Checklist.
